# Assessing imprecision in Cochrane systematic reviews: a comparison of GRADE and Trial Sequential Analysis

**DOI:** 10.1186/s13643-018-0770-1

**Published:** 2018-07-28

**Authors:** Greta Castellini, Matteo Bruschettini, Silvia Gianola, Christian Gluud, Lorenzo Moja

**Affiliations:** 1 Department of Biomedical Sciences for Health, University of Milan, via Pascal 36, 20133 Milan, Italy; 2 Unit of Clinical Epidemiology, IRCCS Galeazzi Orthopedic Institute, via Galeazzi, 4, 20161 Milan, Italy; 3 Department of Paediatrics, Skåne University Hospital, Lund, Sweden; 4 Department of Research & Development, Section for HTA analysis Lund University, Lund, Sweden; 5 Department of Health Sciences Center of Biostatistics for Clinical Epidemiology, University of Milan–Bicocca, Monza, Italy; 6 The Copenhagen Trial Unit, Centre for Clinical Intervention Research, Copenhagen, Denmark

**Keywords:** Review, Meta-analysis, Bias, Confidence intervals, Epidemiologic methods

## Abstract

**Background:**

The evaluation of imprecision is a key dimension of the grading of the confidence in the estimate. Grading of Recommendations Assessment, Development and Evaluation (GRADE) gives recommendations on how to downgrade evidence for imprecision, but authors vary in their use. Trial Sequential Analysis (TSA) has been advocated for a more reliable assessment of imprecision. We aimed to evaluate reporting of and adherence to GRADE and to compare the assessment of imprecision of intervention effects assessed by GRADE and TSA in Cochrane systematic reviews.

**Methods:**

In this cross-sectional study, we included 100 Cochrane reviews irrespective of type of intervention with a key dichotomous outcome meta-analyzed and assessed by GRADE. The methods and results sections of each review were assessed for adequacy of imprecision evaluation. We re-analyzed imprecision following the GRADE Handbook and the TSA Manual.

**Results:**

Overall, only 13.0% of reviews stated the criteria they applied to assess imprecision. The most common dimensions were the 95% width of the confidence intervals and the optimal information size. Review authors downgraded 48.0% of key outcomes due to imprecision. When imprecision was re-analyzed following the GRADE Handbook, 64% of outcomes were downgraded. Agreement between review authors’ assessment and assessment by the authors of this study was moderate (kappa 0.43, 95% confidence interval [CI] 0.23 to 0.58). TSA downgraded 69.0% outcomes due to imprecision. Agreement between review authors’ GRADE assessment and TSA, irrespective of downgrading levels, was moderate (kappa 0.43, 95% CI 0.21 to 0.57). Agreement between our GRADE assessment following the Handbook and TSA was substantial (kappa 0.66, 95% CI 0.49 to 0.79).

**Conclusions:**

In a sample of Cochrane reviews, methods for assessing imprecision were rarely reported. GRADE according to Handbook guidelines and TSA led to more severe judgment of imprecision rather than GRADE adopted by reviews’ authors. Cochrane initiatives to improve adherence to GRADE Handbook are warranted. TSA may transparently assist in such development.

**Electronic supplementary material:**

The online version of this article (10.1186/s13643-018-0770-1) contains supplementary material, which is available to authorized users.

## Background

The clinical sciences attempt to solve uncertainties and disagreements on the effectiveness of interventions by making research findings valid and interpretable. The Grading of Recommendations Assessment, Development and Evaluation (GRADE) system has gained momentum as an internationally recognized framework to assess the quality of evidence systematically and transparently [1]. The GRADE approach suggests evaluating five key dimensions that may affect our confidence in intervention effects: risks of bias (risks of systematic errors), imprecision (risks of random errors), inconsistency, indirectness, and publication bias [2, 3]. Imprecision, along with risk of bias, are the most common domains associated with GRADE downgrading of overall evidence quality or certainty [4].

Systematic reviews employ multiple parameters to evaluate imprecision: accrued sample size, required or optimal information size (OIS) (meta-analytic “sample size”), alpha, beta, confidence intervals of the overall effect, and specified critical margins of “no effect,” “important benefit,” or “important harm” [2]. GRADE combines all components in a simple rule: “If the optimal information size criterion is not met, rate down for imprecision, unless the sample size is very large (at least 2000, and perhaps 4000 patients); if the optimal information size criterion is met and the 95% confidence interval (CI) excludes no effect, do not rate down for imprecision; if the optimal information size criterion is met, and the 95% CI overlaps no effect, rate down for imprecision if the CI fails to exclude important benefit or important harm” [5, 6].

The GRADE rules of thumb are based on broad assumptions and generalities across medical fields. The most relevant advantage is facilitating the trustworthiness of recommendations, enabling users to reflect on the sample as a basis for recommendations. However, rating imprecision in isolation, without a formal evaluation of accrued sample and magnitude of effects (e.g. benefits or harms), would be hazardous [5, 7]. Because random errors are a frequent cause of erroneous estimation of treatment effect, often in small meta-analyses [8], several authors have highlighted the need to adjust the statistical threshold and calculate a required information size in meta-analyses to increase the validity and reliability of its conclusions [9, 10]. Among the techniques that can control for the risk of random error in the context of sparse data [11], Trial Sequential Analysis (TSA) is often used to control for spurious findings [12, 13] and is currently suggested as a potential supplement for a more throughout assessment of imprecision when using the GRADE system [14].

### Objectives

We conducted an empirical assessment of a sample of Cochrane systematic reviews (SRs) in which the focus was imprecision as a threat to validity. We investigated the reporting of and the adherence to GRADE in assessing imprecision, the expected and observed downgrading of evidence, and the reasons for downgrading. Moreover, after having estimated the Cochrane authors’ handling of GRADE and imprecision, we applied ourselves GRADE assessment of imprecision following the GRADE Handbook guidelines [6] and TSA following the TSA Manual [15] to independently replicate the assessments of imprecision.

## Methods

### Search strategy

For this cross-sectional study, Cochrane systematic reviews were sampled from the Cochrane Database of Systematic Reviews [16]. We purposively retrieved 100 reviews in reverse chronological order starting at the time of our search, 23 February 2017. The most current reviews, i.e. the latest published, were selected to ensure inclusion of the most recent publications following the introduction of GRADE [1] and its detailed guidance [2, 3, 5, 6]. The nature of this study was explorative, and no sample size was calculated.

### Eligibility criteria and study selection

Titles and abstracts were screened for eligibility in their chronological order of publication. Full texts were retrieved and evaluated against our inclusion criteria by one investigator. A second investigator checked all eligible records, and a final list was agreed. Cochrane systematic reviews were considered eligible for inclusion if (1) they were reviews of interventions, (2) they meta-analyzed at least two randomized controlled trials (RCTs) for a dichotomous outcome, and (3) the dichotomous outcome was listed in the summary of findings (SoF) table.

We excluded diagnosis/prognosis reviews, studies on health service organization, overviews of systematic reviews and network meta-analyses, and meta-analyses with only uninformative trials (i.e., with no events). The unit of analysis was one outcome for each review: either the primary outcome or the first outcome meta-analyzed and listed in the SoF. We reasoned a priori that this outcome would most likely provide the basis for calculating sample size and orient clinical decision-making.

### Data collection

#### General characteristics and reporting of GRADE in Cochrane reviews

Two investigators independently extracted data from all selected reviews. A third investigator resolved disagreements. We used a standardized ad hoc data collection form that we piloted on the first five Cochrane reviews and then revised according to problems identified. For each review, we sought general information (e.g., author, contact author country, Cochrane review group name, new or updated review, type of intervention—pharmacological or non-pharmacological).

We evaluated what authors reported in the review methods section for assessment of imprecision. In particular, we wanted to determine whether the authors stated they had assessed imprecision and how (e.g., required or optimal information size, benefit/harm thresholds, width of 95% confidence intervals (CI), use of TSA) and in what way they planned to use imprecision assessment (e.g., evidence is downgraded when the required or optimal information size is not reached). We then recorded the grading of imprecision of the outcome selected from the SoF table and the reasons for downgrading. In some cases, we searched other sections of the full-text article for additional information.

#### Adherence to GRADE

We judged whether the review authors adhered to GRADE guidance for downgrading or non-downgrading evidence for imprecision. To determine whether the imprecision evaluation was appropriate (e.g., expected and observed downgrading of evidence), we consulted the instructions for downgrading for imprecision and re-assessed the optimal information size as suggested by the GRADE Handbook guidelines [17]. For each review, we calculated the optimal information size in which we assumed an alpha of 0.05, a beta of 0.20, and an a priori anticipated intervention effect—e.g., risk ratio reduction (RRR) or improvement—defined using the clinically relevant threshold reported by the review authors or, when not stated, the RRR suggested by GRADE authors, as a default threshold of 25% [3], and the control event proportion of the meta-analysis. We used the normogram for events proposed by Guyatt et al. to determine the expected optimal information size [2]. Finally, we determined whether the reviewers incorporated and reported the imprecision assessment in their evaluation of evidence quality.

#### Agreement between GRADE assessment of imprecision and TSA

We evaluated agreement between downgrading of evidence as proposed in the original reviews and those performed by the authors of this article, and that resulting from TSA. For each review, we performed overarching TSAs: for each outcome, we re-analyzed all trials that had been originally included in the meta-analysis. Data were synthesized using the same effect size with its 95% confidence interval (CI) and the same meta-analytic technique (i.e., random effects or fixed effect models), applying the reported statistical heterogeneity (*I*^2^ value). Each trial was sequentially added in the TSA by publication year; this created a series of time points that formed the basis of the cumulative analysis. All TSAs were performed using TSA software (v 0.9.5.5 Beta) [18]. For each review, we calculated the diversity-adjusted required information size (DARIS) using, again, an alpha of 0.05, a beta of 0.20, and a RRR as defined by the review authors or the default threshold of 25%, and the control event proportion. When a random effects model was chosen, between-trial variability was taken into account by adjusting the required information size with the diversity (D^2^) originating from the meta-analysis of trials [19]. The Lan-DeMets trial sequential monitoring boundaries based on O’Brien-Fleming α-spending function was used [13, 20]. The cumulative Z-curve (the series of Z-statistics after each consecutive trial) was calculated and plotted against these monitoring boundaries.

In our primary analysis, we assumed for TSA minimal important or realistic anticipated intervention effects for all outcomes. If, following TSA methods [10], none of the sequential boundaries for benefit, harm, or futility were crossed, imprecision was downgraded by two levels (Additional file 1 reports TSA judgment).

### Data analysis

The data were summarized with descriptive statistics. Absolute and relative frequencies for categorical items and median and interquartile range (IQR) for continuous items were used. We reported adherence to GRADE through figures and agreement between the assessments involving the use of GRADE and TSA in contingency tables, with calculation of Cohen’s kappa [21].

Agreement between GRADE imprecision assessment performed by the review authors and TSA was rated on the ordinal scale as: 0, not downgraded; 1, downgraded by 1 level; 2, downgraded by 2 levels. Moreover, we dichotomized the ordinal scale into “downgraded” and “not downgraded” for imprecision to evaluate the agreement irrespective of level of downgrading. Interpretation of agreement strength (k-values) was made according to the scale devised by Landis and Koch: < 0.00 poor, 0–0.20 slight, 0.21–0.40 fair, 0.41–0.60 moderate, 0.61–0.80 substantial, and 0.81–1.00 almost perfect [22].

Univariable logistic regression was performed to investigate the impact of variables on downgrading for imprecision (dependent variable): Cochrane Group, the country of the contact author, type of intervention, number of patients included in the meta-analysis, heterogeneity among trials, and meta-analysis technique (random effects or fixed effect models). Each method, GRADE assessment of imprecision by the review authors, GRADE performed by the authors of this article, and TSA, was separately assessed and one variable evaluated at a time. The impact of these variables on the agreement between the methods was then tested.

For hypothesis testing, a probability value of < .05 was considered statistically significant. All statistical tests were two-sided. Stata statistical software was used for all statistical analyses [23].

#### Sensitivity analysis

TSAs were replicated using RRRs of (A) 20% and (B) 30%, keeping all other assumptions the same. The concordance between the GRADE judgment on imprecision by the review authors and the TSA assessment was calculated irrespective of the levels of downgrading to determine whether the choice of the anticipated intervention effect affected the agreement.

## Results

### Characteristics of Cochrane reviews

**Fig. 1:**
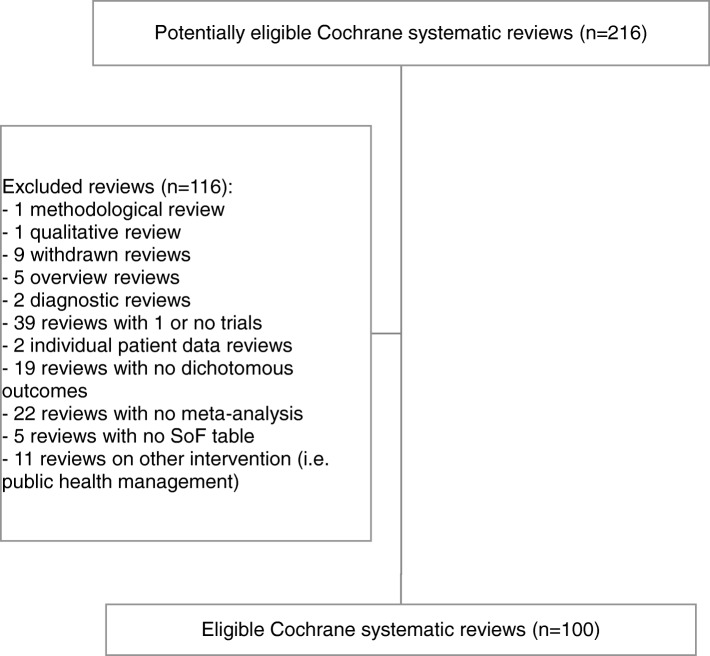
Flow chart of systematic reviews

We included 100 out of 216 potentially eligible Cochrane systematic reviews published in 2017 (issues 1 and 2) and 2016 (issues 12 and 11) of *The Cochrane Library* (Fig. 1), involving 36 (67.9%) out of 53 different Cochrane groups. Figure 1 shows the flow chart of reviews’ selection with reasons for exclusion. Additional file 2 reports included reviews and their main characteristics.

The three most active review groups were pregnancy and childbirth (*n* = 13 reviews), neonatal (*n* = 11 reviews), and heart (*n* = 8 reviews). The corresponding authors were based in the UK (*n* = 28 reviews), Australia (*n* = 15 reviews), or Canada (n = 11 reviews). Sixty-one Cochrane reviews were updates of previous reviews. Table 1 presents the general characteristics of the reviews.

**Table 1:** General characteristics of the 100 Cochrane systematic reviews

Characteristics	Value (no. of reviews)
No. of countries (total no.)	22
Top five countries	
- UK	28
- Australia	15
- Canada	11
- China	7
- Italy	6
No. of Cochrane groups (total no,)	36
Top five Cochrane groups	
- Cochrane Pregnancy and Childbirth Group	13
- Cochrane Neonatal Group	11
- Cochrane Heart Group	8
- Cochrane Airways Group	6
- Cochrane Gynecology and Fertility Group	6
Status of systematic reviews (out of 100)	
- Updated	61
- New	39
Type of intervention (out of 100)	
- Pharmacological	54
- Non-pharmacological	46

Overall, meta-analyses on the selected outcome were performed with a median of five RCTs (IQR, 2 to 9 RCTs; range, 2 to 30 RCTs). Most meta-analyses (81.0%) reported an effect measure expressed as risk ratio, 17.0% used the odds ratio, and only 2.0% reported the risk difference. Half of the SRs (51.0%) achieved statistically significant results according to the naïve 95% CI. The median heterogeneity of the meta-analyses was 12.0% (IQR, 0.0 to 49.0%; range, 0.0 to 98.0%).

### Reporting of GRADE in Cochrane reviews

Nearly all (96.0%) of the reviews referred to GRADE in their methods section (Table 2). Of the four reviews that did not mention GRADE but performed it, two presented some information in the discussion. Very few (13.0%) of the reviews that graded the evidence reported the criteria they applied to assess imprecision. The most common imprecision components were width of 95% CI (8.0%) and optimal information size referred to participants (4.0%). Ten reviews combined at least two criteria to assess imprecision. Only two reviews reported a comprehensive list of reasons behind imprecision judgment, thus allowing for full replication. Two other reviews planned and conducted a TSA. Neither the publisher Cochrane Group nor the country of the contact author influenced the reporting of imprecision assessment (univariable logistic regressions, respectively: Cochrane Group, *p* = 0.716; country of contact author, *p* = 0.782).

**Table 2:** Approaches to assessment of imprecision and formal quantitative analyses of imprecision in Cochrane systematic reviews. Values are numbers

	Reported (no. of reviews out of 100)
Methods section	
Reviewers carried out GRADE assessment	96
Criteria considered for assessing imprecision?	13
- Width of 95% confidence interval	8
- Optimal information size—no. of participants	4
- Optimal information size—no. of events	1
- Threshold for benefit or harm	1
- Trial Sequential Analysis	2
Results section	
Optimal information size—no. of events	2
Optimal information size—no. of participants	8
Thresholds for benefit or harm	15
Trial Sequential Analysis	2

The quality of the meta-analyzed dichotomous outcomes was often graded as low (41.0%), with few outcomes reaching high quality (9.0%). Few reviews clearly stated on which criteria their assessment of imprecision was based. However, lack of details on how imprecision was assessed did not prevent the systematic reviewers to evaluate it, completing the SoF. Overall, almost half of the outcomes (48.0%) were downgraded for imprecision, with only six reviews downgrading imprecision by two levels. The most frequent reasons for downgrading due to imprecision were low number of events or small sample size (26.0%) and wide 95% CIs (25.0%). Six outcomes were downgraded due to imprecision, but no reason was reported in the SoF tables or full-text.

### Adherence to GRADE Handbook instructions

**Fig. 2:**
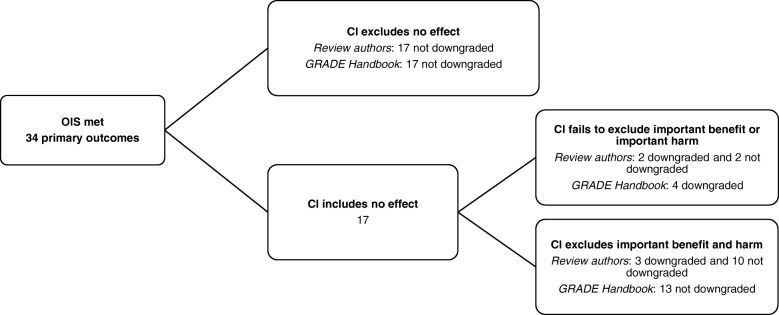
Primary outcomes that met the OIS—number of events: comparing GRADE assessment of imprecision carried out by review authors with GRADE carried out by the authors of this article following GRADE Handbook guidelines

**Fig. 3:**
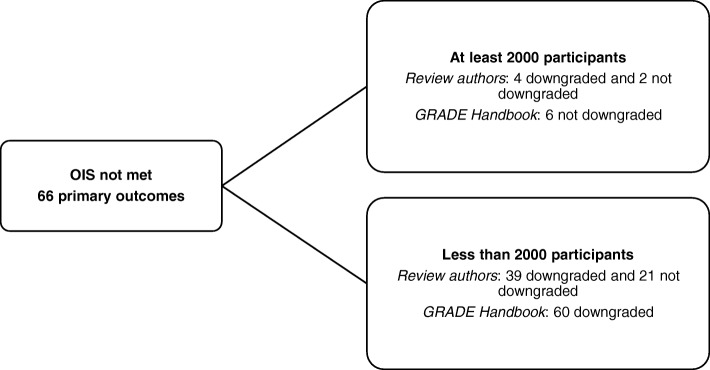
Primary outcomes that did not meet the OIS—number of events: comparing GRADE assessment of imprecision carried out by review authors with GRADE carried out by the authors of this article following GRADE Handbook guidelines

When the authors of this article followed the GRADE Handbook instructions on how to replicate assessment and evaluate adherence, 64 outcomes were downgraded due to imprecision. Sixty-six did not meet the OIS for events. Overall, in 30.0% of reviews, judgment of outcomes differed between the review authors and the authors of this article who followed the GRADE Handbook (Figs. 2 and 3). Cohen’s kappa coefficient between the grading of imprecision as proposed by the original authors and as re-analyzed by us following the GRADE Handbook was 0.43 (95% CI 0.23 to 0.58), which expressed moderate strength of agreement.

### Imprecision by TSA

The anticipated intervention effect was reported only by 12 reviews. For the other reviews, we adopted a 25% RRR to calculate the required information size. Overall, 69 outcomes were downgraded due to imprecision by applying TSA (downgrading by two levels since the anticipated intervention effect was assumed as being realistic). Indeed, five more outcomes were downgraded for imprecision by applying TSA as compared to using the GRADE Handbook instructions. The required information size was reached by 17 meta-analyses (17.0%). In the remaining 83.0%, the median number of participants needed to reach the required information size was 4187 (IQR, 1467 to 11,104 participants).

### Agreement between GRADE by review authors and TSA

Weighted Cohen’s kappa coefficient showing agreement between GRADE performed by review authors and TSA was 0.20 (95% CI 0.11 to 0.30). The coefficient expressed slight agreement (Table 3).

**Table 3:** Concordance in downgrading due to imprecision by 1 and 2 levels between GRADE carried out by the review authors and with TSA

	TSA			
	Not downgraded	Downgraded by 1 level	Downgraded by 2 levels	Total
GRADE by review authors				
Not downgraded	27	0	25	52
Downgraded by 1 level	3	0	39	42
Downgraded by 2 levels	1	0	5	6
Total	31	0	69	100

Considering only the outcomes downgraded or not downgraded due to imprecision, irrespective of levels, unweighted Cohen’s kappa coefficient was 0.43 (95% CI 0.21 to 0.57), expressing moderate strength of agreement (Table 4).

**Table 4:** Concordance in downgrading due to imprecision between GRADE carried out by review authors, by authors of this article, and with TSA

	TSA		
	Not downgraded	Downgraded	Total
GRADE by review authors			
Not downgraded	27	25	52
Downgraded	4	44	48
Total	31	69	100
GRADE by the authors of this article			
Not downgraded	26	10	36
Downgraded	5	59	64
Total	31	69	100

### Agreement between GRADE by authors of this article and TSA

The imprecision evaluated by the authors of this article following the GRADE Handbook guidelines and by TSA was similar: unweighted Cohen’s kappa coefficient was 0.66 (95% CI 0.49 to 0.79), expressing substantial agreement (Table 4)*.*

### Results of logistic regression analyses

In the univariable logistic regression analysis, the type of the intervention (GRADE by review authors: *p* = 0.65; TSA *p* = 0.78), the number of patients included in the meta-analysis (GRADE by the review authors: *p* = 0.08; TSA *p* = 0.07), the heterogeneity (GRADE by review authors: *p* = 0.12; TSA *p* = 0.38), and the meta-analysis technique (random effects or fixed effect models) (GRADE by review authors: *p* = 0.86; TSA *p* = 0.29) were not associated with downgrading due to imprecision.

When GRADE assessment of imprecision carried out by the review authors was compared to TSA assessment and GRADE replicated by the authors of this article according to the Handbook guidelines, it seemed that the meta-analytic model (random or fixed effect) might influence the agreement in both cases (*p* = 0.04 and *p* = 0.09, respectively), whereas the number of patients might influence only agreement between GRADE by the authors of this article and TSA (*p* = 0.01).

Logistic regression tables are added as Additional file 3.

### Sensitivity analysis

TSAs with an anticipated intervention effect of 30% RRR revealed 60.0% of the SRs downgraded due to imprecision. When the anticipated intervention effect was lowered to 20% RRR, the percentage of SRs downgraded due to imprecision increased to 73.0%. Cohen’s kappa coefficients, expressing downgrading or not due to imprecision, irrespectively of the downgrading levels (by 1 or 2), did not change much: coefficients 0.43 (95% CI, 0.22 to 0.58) and 0.43 (95% CI, 0.20 to 0.56), respectively.

## Discussion

Given the implications of imprecision evaluation for recommending health care interventions as standard of care or cautionary noting that additional clinical trials are warranted, it is expected that imprecision be transparently evaluated and reported. There remains ample room for improvement, however. Almost half of the outcomes were downgraded due to imprecision, but only about 1 in 10 reviews reported the criteria to downgrade imprecision. The width of 95% of confidence intervals and the number of study participants were the most common criteria to infer downgrading due to imprecision. One third of the conclusions that did not downgrade the evidence for imprecision would have been contradicted based on GRADE assessment of imprecision following the GRADE Handbook or TSA if these methods had been applied. This was mainly because GRADE evaluation by the review authors was more lenient and also because the number of patients included in the meta-analyses was often insufficient to make any definitive conclusion.

The GRADE approach and TSA have different connotations that might influence the judgment process. While GRADE is defined as “a semi quantitative approach that encompasses imprecision besides the other certainty domains” and has intrinsic subjectivity [5], TSA can be viewed as a purely quantitative and objective approach [10]. Despite their differences, the agreement between the two approaches was substantial. Nonetheless, the two methods give different weight to the extent of imprecision. TSA tended toward more severe judgment, while GRADE seemed to be more easily applied by the review authors, resulting in overestimation of the certainty of evidence. When imprecision is rated as negligible (i.e., the true effect plausibly lies within the 95% CI), it is more likely that the effect estimates can be trusted and evidence quality rated highly. TSA will allow the reader to gauge the extent of confidence on imprecision of primary results of meta-analyses, though it might also be perceived as too radical by some. With a GRADE approach, reviewers’ decisions on downgrading are more open to subjective decisions. To guide such decisions, further research is warranted on optimal information size and of different types of interventions under different circumstances.

### Strengths and weaknesses of the study

This study has several limitations. We included only Cochrane reviews of health care interventions, a fairly homogeneous but partial sample of systematic reviews published in the medical literature. Reviews published in other medical journals might provide different results. However, since Cochrane strongly encourages the use of the GRADE approach, including imprecision, it seems implausible that other journals would perform better. Our analyses are valid for pooled results and restricted to dichotomous outcomes, limiting the generalizability of our results. However, in the medical literature, two thirds of primary outcomes reported by systematic reviews are dichotomous [24]. Besides, calculation and definition of a clinical threshold for continuous outcomes, i.e., the minimal important differences or minimal detectable changes, remain ambiguous and unclear [25, 26]. Furthermore, our logistic regression results may be under-powered to detect any significant factor, due to the limited number of studies included in the analysis.

While our analysis was protected against confounding by disease area and type of intervention because it is based on a sample of meta-analyses irrespective of interventions, it may still have been confounded by other meta-analysis characteristics. Given the wide diversity of the studies included, the quantitative results are suggestive and might change in the future when GRADE and Cochrane propose methods and policies that strengthen imprecision assessment. Furthermore, imprecision assessment may vary between research areas (i.e., type of interventions), becoming speculative in fields where trials and reviews are too small to permit exploration of imprecision.

When we evaluated the conclusiveness of evidence by TSA in studies where the anticipated treatment effect was not reported, we could not directly assess the authors’ assumptions on relevant intervention effects. We chose one arbitrary hypothesis when we recalculated the required information size based on a 25% relative risk reduction or improvement, which might be unrealistic for some outcomes. Nevertheless, the proportion of primary outcomes downgraded for imprecision did not change substantially when we applied different thresholds for benefits in our sensitivity analyses. Moreover, we only used a two-level downgrading approach when our TSA did not show benefit, harm, or futility. By, e.g., employing the Trial Sequential Analysis-adjusted confidence intervals, this simple approach could have been refined.

As well, our results are based on our subjective application of the GRADE approach following the GRADE Handbook, which would then influence the assessments we made between the methods.

Despite these limitations, this project aspires to be a step toward optimized assessment and interpretation of the certainty of evidence regarding imprecision. Recently, evidence synthesis has evolved into a dynamic process. A new concept of systematic reviews, *living systematic reviews*, has been introduced [27]*.* In this context, TSA has been suggested as a valid method to assess constantly updated evidence [12] since it can offer constantly updating of optimal information size as new trials are added. This dynamicity could affect the imprecision domain, with assessment more closely related to what has been reached for a specific condition, outcome, and intervention at a certain time point.

### Implications for systematic reviewers

As part of their mandate to identify potentially effective interventions, systematic reviewers should include precision as a key dimension to evaluate, particularly in situations where uncertainty about the ratio between benefits and harms is high and where new trial data may influence the summary judgment of the review [28]. More detailed guidance from PRISMA and PRISMA-P could facilitate the analysis and reporting of imprecision [29, 30]. There is room for better standardization of approaches and inclusion of quantitative methods, such as TSA, to formally evaluate imprecision.

### Implications for clinicians

Imprecision assessment seems to be based on GRADEing criteria that vary considerably in meaning, value, or boundaries depending on context or conditions. Incomplete or vague imprecision assessment supports the natural tendency to simplify a review’s findings about an intervention as positive or negative and to over-rely on *P* values [31]. Instead, clinicians using evidence to orient their practice should interpret imprecision as a dynamic and often uncertain dimension that requires thorough examination of all of the evidence, including size of the trials, number of participants with outcomes, and heterogeneity (diversity) across trials. TSA offers the means to model heterogeneity and estimate optimal information size based on different heterogeneity thresholds.

### Future research

Further research is needed to determine whether accurate assessment of imprecision might change the clinicians’ perception about the definitive effectiveness of interventions. It would be beneficial to develop an international database of prospectively updated TSAs for all health interventions, where people can easily consult the progress of research. This might also help to diminish “the butterfly behavior of researchers” from moving onto the next research question before the previous quest has been fully exploited [32].

## Conclusions

A significant lack of reporting of and adherence to GRADE was observed in Cochrane systematic reviews. Stricter adherence to GRADE Handbook guidelines and/or adoption of TSA would have led to more frequent downgrading of the quality of evidence. Our findings reiterate the need for a more reliable application of GRADE in Cochrane and non-Cochrane reviews for the assessment of imprecision. The reasons for downgrading should be defined following a more structured reporting system. Initiatives to improve adherence to GRADE indications are warranted to ensure high-quality systematic reviews. Cochrane groups, peer reviewers, and editors need to be more supportive during the GRADE assessment process as it is still difficult to apply and requires a structured reporting policy to ensure transparency and intelligibility in the process.

## Additional files

Additional file 1: Trial Sequential Analysis as supplement of GRADE assessment for imprecision. (DOCX 13 kb)

Additional file 2: Cochrane systematic reviews with general characteristics. (DOCX 24 kb)

Additional file 3: Logistic regressions results. (DOCX 273 kb)

## Abbreviations

CI: Confidence interval
GRADE: Grading of Recommendations Assessment Development and Evaluation
IQR: Interquartile range
OIS: Optimal information size
RCT: Randomized controlled trial
RRR: Relative risk reduction
SoF: Summary of findings
SRs: Systematic reviews
TSA: Trial Sequential Analysis

## References

[1] Guyatt GH, Oxman AD, Vist GE, Kunz R, Falck-Ytter Y, Alonso-Coello P, Schunemann HJ (2008). GRADE: an emerging consensus on rating quality of evidence and strength of recommendations. BMJ.

[2] Guyatt GH, Oxman AD, Kunz R, Brozek J, Alonso-Coello P, Rind D, Devereaux PJ, Montori VM, Freyschuss B, Vist G (2011). GRADE guidelines 6. Rating the quality of evidence--imprecision. J Clin Epidemiol.

[3] Guyatt G, Oxman AD, Akl EA, Kunz R, Vist G, Brozek J, Norris S, Falck-Ytter Y, Glasziou P, DeBeer H (2011). GRADE guidelines: 1. Introduction-GRADE evidence profiles and summary of findings tables. J Clin Epidemiol.

[4] Pandis N, Fleming PS, Worthington H, Salanti G (2015). The quality of the evidence according to GRADE is predominantly low or very low in oral health systematic reviews. PLoS One.

[5] Schunemann HJ (2016). Interpreting GRADE's levels of certainty or quality of the evidence: GRADE for statisticians, considering review information size or less emphasis on imprecision?. J Clin Epidemiol.

[6] GRADE handbook for grading quality of evidence and strength of recommendations. [database on the Internet]. The GRADE Working Group 2013. Available from: guidelinedevelopment.org/handbook. Accessed Jan 2017.

[7] Anttila S, Persson J, Vareman N, Sahlin NE (2016). Conclusiveness resolves the conflict between quality of evidence and imprecision in GRADE. J Clin Epidemiol.

[8] Imberger G, Thorlund K, Gluud C, Wetterslev J. False-positive findings in Cochrane meta-analyses with and without application of Trial Sequential Analysis: an empirical review. BMJ Open. 2016;6:e011890. 10.1136/bmjopen-2016-011890. 10.1136/bmjopen-2016-011890 PMC4985805 27519923

[9] Thorlund K, Imberger G, Walsh M, Chu R, Gluud C, Wetterslev J, Guyatt G, Devereaux PJ, Thabane L (2011). The number of patients and events required to limit the risk of overestimation of intervention effects in meta-analysis—a simulation study. PLoS One.

[10] Jakobsen JC, Wetterslev J, Winkel P, Lange T, Gluud C (2014). Thresholds for statistical and clinical significance in systematic reviews with meta-analytic methods. BMC Med Res Methodol.

[11] Higgins JP, Whitehead A, Simmonds M (2011). Sequential methods for random-effects meta-analysis. Stat Med.

[12] Simmonds M, Salanti G, McKenzie J, Elliott J, Living Systematic Review N (2017). Living systematic reviews: 3. Statistical methods for updating meta-analyses. J Clin Epidemiol.

[13] Wetterslev J, Jakobsen JC, Gluud C. Trial Sequential Analysis in systematic reviews with meta-analysis. BMC Med Res Methodol. 2017;17:39. 10.1186/s12874-017-0315-7 PMC5397700 28264661

[14] Jakobsen JC, Gluud C, Winkel P, Lange T, Wetterslev J (2014). The thresholds for statistical and clinical significance—a five-step procedure for evaluation of intervention effects in randomised clinical trials. BMC Med Res Methodol.

[15] Thorlund K, Wetterslev J, Brok J, Imberger G, Gluud G. Trial Sequential Analysis (TSA) manual. Copenaghen; 2011. Available from http://www.ctu.dk/tsa/. Accessed Jan 2017.

[16] Cochrane Library [database on the Internet]. Wiley Online Library. Available from: http://onlinelibrary.wiley.com/cochranelibrary/search. Accessed Feb 2017.

[17] Chapter 5.2.4.2 Imprecision in in systematic reviews in Schünemann H BJ, Guyatt G, Oxman A, editors. GRADE handbook for grading quality of evidence and strength of recommendations. Updated October 2013. The GRADE Working Group, 2013. Available from http://guidelinedevelopment.org/handbook. Accessed Jan 2017.

[18] TSA software. 0.9 beta ed. Copenhagen Trial Unit, Centre for Clinical Intervention Research, Copenhagen, Denmark2011. Available from http://www.ctu.dk/tsa/. Accessed Apr 2017.

[19] Wetterslev J, Thorlund K, Brok J, Gluud C (2009). Estimating required information size by quantifying diversity in random-effects model meta-analyses. BMC Med Res Methodol.

[20] Wetterslev J, Thorlund K, Brok J, Gluud C (2008). Trial Sequential Analysis may establish when firm evidence is reached in cumulative meta-analysis. J Clin Epidemiol..

[21] Watson PF, Petrie A (2010). Method agreement analysis: a review of correct methodology. Theriogenology.

[22] Landis JR, Koch GG (1977). The measurement of observer agreement for categorical data. Biometrics.

[23] StataCorp (2003). Stata Statistical Software: Release 8.

[24] Page MJ, Shamseer L, Altman DG, Tetzlaff J, Sampson M, Tricco AC, Catala-Lopez F, Li L, Reid EK, Sarkis-Onofre R, Moher D (2016). Epidemiology and reporting characteristics of systematic reviews of biomedical research: a cross-sectional study. PLoS Med.

[25] Copay AG, Subach BR, Glassman SD, Polly DW, Schuler TC (2007). Understanding the minimum clinically important difference: a review of concepts and methods. Spine J.

[26] Armijo-Olivo S, Warren S, Fuentes J, Magee DJ (2011). Clinical relevance vs. statistical significance: using neck outcomes in patients with temporomandibular disorders as an example. Man Ther.

[27] Elliott JH, Synnot A, Turner T, Simmonds M, Akl EA, McDonald S, Salanti G, Meerpohl J, MacLehose H, Hilton J (2017). Living systematic review: 1. Introduction-the why, what, when, and how. J Clin Epidemiol.

[28] Riva N, Puljak L, Moja L, Ageno W, Schunemann H, Magrini N, Squizzato A (2017). Multiple overlapping systematic reviews facilitate the origin of disputes: the case of thrombolytic therapy for pulmonary embolism. J Clin Epidemiol..

[29] Moher D, Liberati A, Tetzlaff J, Altman DG, Group P (2009). Preferred reporting items for systematic reviews and meta-analyses: the PRISMA statement. PLoS Med.

[30] Moher D, Shamseer L, Clarke M, Ghersi D, Liberati A, Petticrew M, Shekelle P, Stewart LA, Group P-P. Preferred reporting items for systematic review and meta-analysis protocols (PRISMA-P) 2015 statement. Syst Rev. 2015;4(1). 10.1186/2046-4053-4-1 PMC4320440 25554246

[31] Pocock SJ, Stone GW (2016). The primary outcome is positive—is that good enough?. N Engl J Med.

[32] Liberati A (2004). An unfinished trip through uncertainties. BMJ.

