# Supplementary material for: Assessing imprecision in Cochrane systematic reviews: a comparison of GRADE and Trial Sequential Analysis
Source: Syst Rev. 2018 Jul 28;7:110. doi: 10.1186/s13643-018-0770-1 (PMC6064621; doi:10.1186/s13643-018-0770-1)
Supplement: Supplementary file 1 — Trial Sequential Analysis as supplement of GRADE assessment for imprecision. (DOCX 13 kb) [file 13643_2018_770_MOESM1_ESM.docx]

**Additional file 1**

**Trial Sequential Analysis as supplement of GRADE assessment for ‘imprecision’**

| **Trial Sequential Analysis** | **Assessment of imprecision** |
| --- | --- |
| If none of the sequential boundaries for benefit, harm, or futility are crossed [by the cumulative Z curve] and the anticipated intervention effect is realistic. | The evidence should be downgraded two levels of quality according to imprecision. |
| If one of the boundaries for benefit, harm, or futility are crossed and the anticipated intervention effect is realistic. | The evidence should not be downgraded according to imprecision. |
| If the anticipated intervention effect is considered unrealistic. | The Trial Sequential Analysis should be repeated using the limit of the confidence interval, closest to zero effect from the traditional meta-analysis as the anticipated intervention effect. If the sequential boundaries are crossed [by the cumulative Z curve] then the level of evidence should not be downgraded. If the sequential boundaries are not crossed, the trial sequential analysis should be repeated, this time, using the intervention effect estimate from the meta-analysis as the anticipated intervention effect. If the sequential boundaries are crossed [by the cumulative Z curve], then the evidence should be downgraded one level of quality; if the sequential boundaries are not crossed [by the cumulative Z curve], then the evidence should be downgraded two levels of quality. |

Table based upon: Jakobsen JC, Wetterslev J, Winkel P, Lange T, Gluud C. **Thresholds for statistical and clinical significance in systematic reviews with meta-analytic methods.** *BMC Medical Research Methodology* 2014, 14:120.
