# Supplementary material for: Assessing imprecision in Cochrane systematic reviews: a comparison of GRADE and Trial Sequential Analysis
Source: Syst Rev. 2018 Jul 28;7:110. doi: 10.1186/s13643-018-0770-1 (PMC6064621; doi:10.1186/s13643-018-0770-1)
Supplement: Supplementary file 2 — Cochrane systematic reviews with general characteristics. (DOCX 24 kb) [file 13643_2018_770_MOESM2_ESM.docx]

| Additional file 2. Included Cochrane systematic reviews. | | | | |
| --- | --- | --- | --- | --- |
| N° | First author’s name | Contact author’s country | New(N)/  Update(U) | Cochrane Group |
| 1 | Kim | USA | N | Cochrane Eyes and Vision Group |
| 2 | Offringa | Canada | U | Cochrane Epilepsy Group |
| 3 | Gimzewska | UK | U | Cochrane Vascular Group |
| 4 | Gowing | Australia | U | Cochrane Drugs and Alcohol Group. |
| 5 | Coppolino | Italy | N | Cochrane Hypertension Group |
| 6 | Johnston | Canada | U | Cochrane Neonatal Group |
| 7 | Urquhart | UK | U | Cochrane Pregnancy and Childbirth Group |
| 8 | Chamberlain | Australia | U | Cochrane Pregnancy and Childbirth Group |
| 9 | Robertson | UK | U | Cochrane Vascular Group |
| 10 | Eke | USA | N | Cochrane Hepato-Biliary Group |
| 11 | Horita | Japan | N | Cochrane Airways Group |
| 12 | Molakatalla | Australia | N | Cochrane Pregnancy and Childbirth Group |
| 13 | Robertson | UK | U | Cochrane Vascular Group |
| 14 | Risom | Denmark | N | Cochrane Heart Group |
| 15 | Salazar | Peru | N | Cochrane Heart Group |
| 16 | Jiang | China | U | Cochrane Pregnancy and Childbirth Group |
| 17 | Sun | China | N | Cochrane Colorectal Cancer Group |
| 18 | van Zuuren | Netherlands | N | Cochrane Skin Group |
| 19 | Foster | Australia | N | Cochrane Neonatal Group |
| 20 | Alfirevic | UK | U | Cochrane Pregnancy and Childbirth Group |
| 21 | Crossingham | UK | N | Cochrane Airways Group |
| 22 | Lemyre | Canada | U | Cochrane Neonatal Group |
| 23 | Østerås | Norway | N | Cochrane Musculoskeletal Group |
| 24 | Borthwick | UK | U | Cochrane Anaesthesia, Critical and Emergency Care Group |
| 25 | Kim | Switzerland | U | Cochrane Pregnancy and Childbirth Group |
| 26 | Ng | Canada | U | Cochrane Neonatal Group |
| 27 | Onland | Netherlands | N | Cochrane Neonatal Group |
| 28 | Mocellin | Italy | U | Cochrane Colorectal Cancer Group |
| 29 | Estcourt | UK | N | Cochrane Haematological Malignancies Group |
| 30 | Wardle | UK | N | Cochrane Vascular Group |
| 31 | Devane | Ireland | U | Cochrane Pregnancy and Childbirth Group |
| 32 | Kyrgiou | UK | U | Cochrane Gynaecological, Neuro-oncology and Orphan Cancer Group |
| 33 | Bordewijk | Netherlands | N | Cochrane Gynaecology and Fertility Group |
| 34 | Walters | Australia | U | Cochrane Airways Group |
| 35 | Ng | UK | U | Cochrane Neonatal Group |
| 36 | Kietpeerakool | Thailand | N | Cochrane Gynaecological, Neuro-oncology and Orphan Cancer Group |
| 37 | Wiysonge | South Africa | U | Cochrane Hypertension Group |
| 38 | Andriolo | Brazil | N | Cochrane Anaesthesia, Critical and Emergency Care Group |
| 39 | Estcourt | UK | U | Cochrane Cystic Fibrosis and Genetic Disorders Group |
| 40 | Marjoribanks | New Zeland | U | Cochrane Gynaecology and Fertility Group |
| 41 | Cluver | South Africa | N | Cochrane Pregnancy and Childbirth Group |
| 42 | Derry | UK | U | Cochrane Pain, Palliative and Supportive Care Group |
| 43 | Garjón | Spain | N | Cochrane Hypertension Group |
| 44 | Derry | UK | U | Cochrane Pain, Palliative and Supportive Care Group |
| 45 | Wieland | USA | N | Cochrane Back and Neck Group |
| 46 | Kirkland | Canada | N | Cochrane Airways Group |
| 47 | Hill | New Zealand | N | Cochrane Kidney and Transplant Group |
| 48 | Abbott | UK | U | Cochrane Developmental, Psychosocial and Learning Problems Group |
| 49 | Hahn | Australia | N | Cochrane Kidney and Transplant Group |
| 50 | Yao | China | U | Cochrane Colorectal Cancer Group |
| 51 | Dieterich | Italy | U | Cochrane Schizophrenia Group |
| 52 | Barrington | Canada | U | Cochrane Neonatal Group |
| 53 | Shah | Canada | U | Cochrane Neonatal Group |
| 54 | Middleton | Australia | U | Cochrane Pregnancy and Childbirth Group |
| 55 | Bromham | UK | N | Cochrane Breast Cancer Group |
| 56 | Tieu | Australia | U | Cochrane Pregnancy and Childbirth Group |
| 57 | Lee | Australia | N | Cochrane Breast Cancer Group |
| 58 | Barrington | Canada | U | Cochrane Neonatal Group |
| 59 | Fisher | UK | U | Cochrane Heart Group |
| 60 | Chang | Canada | N | Cochrane ENT Group |
| 61 | Ma | China | U | Cochrane Injuries Group |
| 62 | McLellan | UK | N | Cochrane Heart Group |
| 63 | van Driel | Australia | U | Cochrane Heart Group |
| 64 | Lazzerini | Italy | U | Cochrane Infectious Diseases Group |
| 65 | Baldwin | UK | N | Cochrane Metabolic and Endocrine Disorders Group |
| 66 | Larun | Norway | U | Cochrane Common Mental Disorders Group |
| 67 | Cabello | Spain | U | Cochrane Heart Group |
| 68 | Howcroft | Australia | U | Cochrane Airways Group |
| 69 | Ma | China | N | Cochrane Oral Health Group |
| 70 | Brown | New Zealand | U | Cochrane Gynaecology and Fertility Group |
| 71 | Lemyre | Canada | U | Cochrane Neonatal Group |
| 72 | Westby | UK | N | Cochrane Wounds Group |
| 73 | Zhu | China | N | Cochrane Eyes and Vision Group |
| 74 | Rattehalli | Australia | U | Cochrane Schizophrenia Group |
| 75 | Brown | New Zealand | U | Cochrane Gynaecology and Fertility Group |
| 76 | Gregorio | Philippines | U | Cochrane Infectious Diseases Group |
| 77 | de Silva | UK | U | Cochrane Eyes and Vision Group |
| 78 | Mbuagbaw | Cameroon | U | Cochrane Infectious Diseases Group |
| 79 | Stewart | UK | U | Cochrane Incontinence Group |
| 80 | Puhan | Switzerland | U | Cochrane Airways Group |
| 81 | Lumbiganon | Thailand | U | Cochrane Pregnancy and Childbirth Group |
| 82 | Rao | Australia | U | Cochrane Neonatal Group |
| 83 | Ziganshina | Russian Federation | U | Cochrane Stroke Group |
| 84 | Ghosh | UK | U | Cochrane Pregnancy and Childbirth Group |
| 85 | Romano | UK | N | Cochrane Eyes and Vision Group |
| 86 | Manfredi | Italy | U | Cochrane Oral Health Group |
| 87 | Avenell | UK | U | Cochrane Bone, Joint and Muscle Trauma Group |
| 88 | Maher | Australia | U | Cochrane Gynaecology and Fertility Group |
| 89 | Bhatt | Australia | N | Cochrane Eyes and Vision Group |
| 90 | Tang | China | U | Cochrane Gynaecology and Fertility Group |
| 91 | Mead | UK | N | Cochrane Metabolic and Endocrine Disorders Group |
| 92 | Paravastu | UK | N | Cochrane Vascular Group |
| 93 | Izquierdo-Palomares | Spain | N | Cochrane Heart Group |
| 94 | Moore | USA | U | Cochrane Pregnancy and Childbirth Group |
| 95 | Jeffery | New Zealand | U | Cochrane Colorectal Cancer Group |
| 96 | Wang | Germany | U | Cochrane Schizophrenia Group |
| 97 | La Mantia | Italy | U | Cochrane Multiple Sclerosis and Rare Diseases of the CNS Group |
| 98 | Apollonio | USA | U | Cochrane Tobacco Addiction Group |
| 99 | Nyong | Canada | N | Cochrane Heart Group |
| 100 | Warttig | UK | N | Cochrane Anaesthesia, Critical and Emergency Care Group. |
