# Supplementary material for: Assessing imprecision in Cochrane systematic reviews: a comparison of GRADE and Trial Sequential Analysis
Source: Syst Rev. 2018 Jul 28;7:110. doi: 10.1186/s13643-018-0770-1 (PMC6064621; doi:10.1186/s13643-018-0770-1)

**Additional file 3. Logistic regressions results.**

**1. GRADE by review authors**


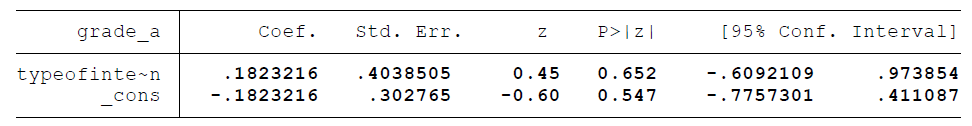
1.1 Type of intervention – pharmacological or not pharmacological


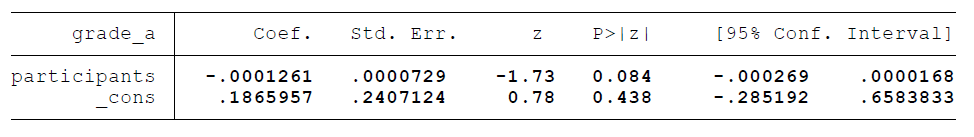
1.2 Number of patients included in the meta-analysis


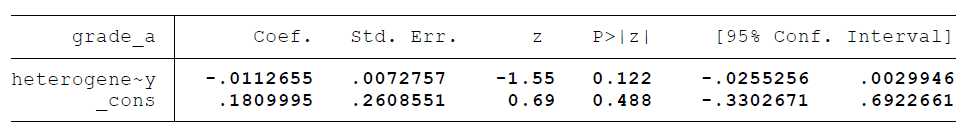
1.3 Heterogeneity


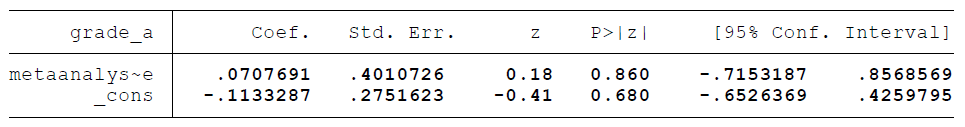
1.4 Meta-analysis technique

**2. Trial Sequential Analysis**


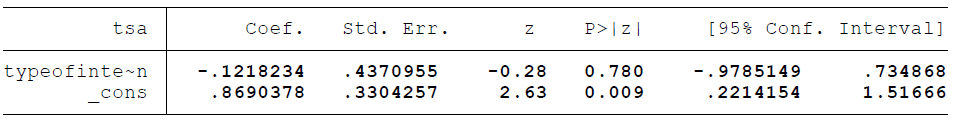
2.1 Type of intervention – pharmacological or not pharmacological

2.2 Number of patients included in the meta-analysis


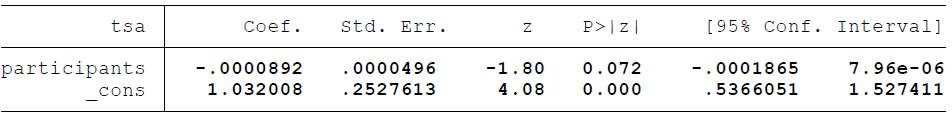


2.3 Heterogeneity


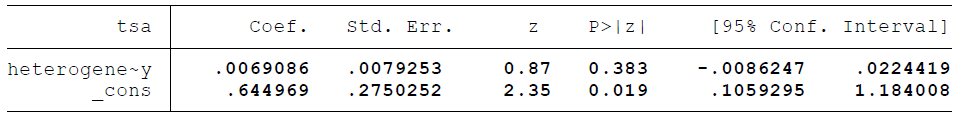


2.4 Meta-analysis technique


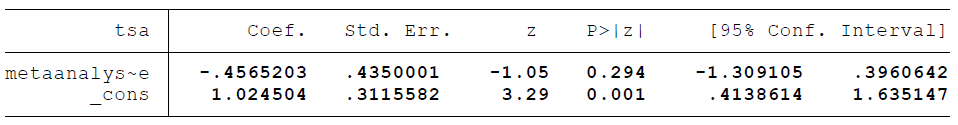


**3. Concordance between GRADE by review authors and TSA**


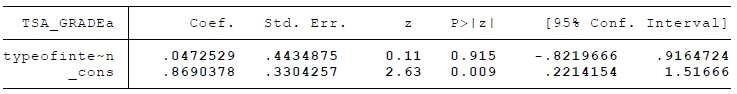
3.1 Type of intervention – pharmacological or not pharmacological


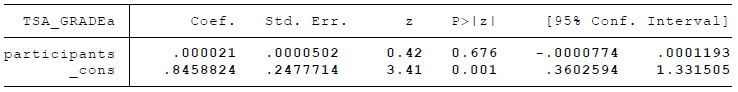
3.2 Number of patients included in the meta-analysis


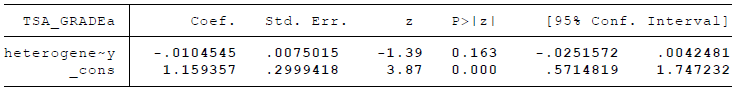
3.3 Heterogeneity

3.4 Meta-analysis technique


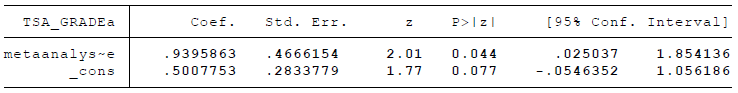


**4. Concordance between GRADE by authors of this article according to the Handbook and TSA**


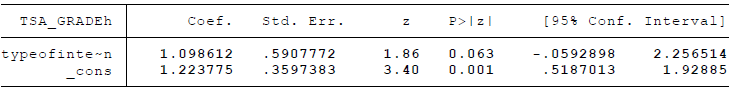
4.1 Type of intervention – pharmacological or not pharmacological


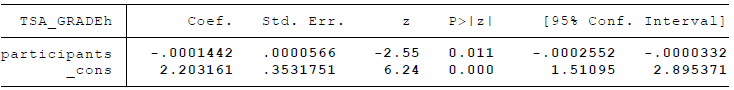
4.2 Number of patients included in the meta-analysis


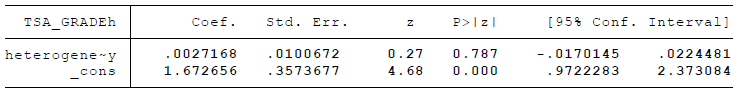
4.3 Heterogeneity


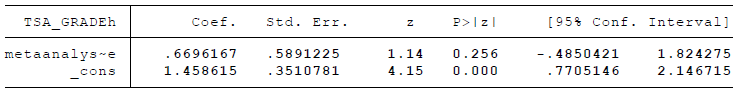
4.4 Meta-analysis technique

**5. Concordance between GRADE by authors of this article according to the Handbook and GRADE by review authors**

5.1 Type of intervention – pharmacological or not pharmacological


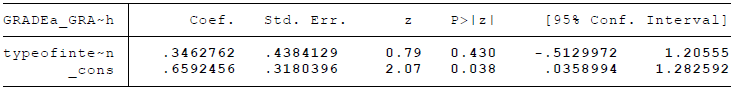


5.2 Number of patients included in the meta-analysis


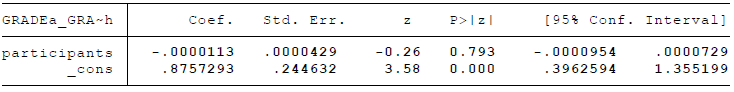


5.3 Heterogeneity


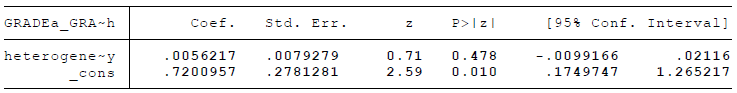


5.4 Meta-analysis technique


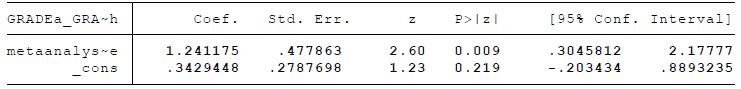

Supplement: Supplementary file 3 — Logistic regressions results. (DOCX 273 kb) [file 13643_2018_770_MOESM3_ESM.docx]
